# Prevalence of Language Delay in Children Under Five Years of Age in Taif, Saudi Arabia

**DOI:** 10.7759/cureus.86960

**Published:** 2025-06-29

**Authors:** Samer A Alzahrani, Sameer R Alharthi, Shahad A Alamri, Noor M Saklou, Shatha F Alharthi, Maram Alayli, Shahad H Alraddadi

**Affiliations:** 1 Department of Pediatrics, Albaha University, Albaha, SAU; 2 Department of Pediatrics, Taif Armed Forces Hospital, Taif, SAU; 3 Department of Medicine, College of Medicine, Taif University, Taif, SAU; 4 Department of Speech-Language Pathology, Taif Armed Forces Hospital, Taif, SAU

**Keywords:** ages and stages questionnaire score, communication, development, language delay, milestones

## Abstract

Background: Developmental delay occurs when a child does not achieve expected milestones, with language delay being a common concern. This is characterized by a considerably slower rate of speech and language development compared to peers.

Aim: The aim of this study is to determine the prevalence of language delay among children under five years of age in Taif, Saudi Arabia, and to explore related factors.

Methods: This cross-sectional study involved 400 participants under the age of five. Data were collected through a hospital-based questionnaire, using Ages and Stages Questionnaire (ASQ) scores specific to each age group. The collected data were entered and analyzed using IBM SPSS Statistics for Windows, Version 21 (Released 2012; IBM Corp., Armonk, New York). All statistical analyses were two-tailed, with an alpha level set at 0.05.

Results: The highest proportion (100%) of children demonstrating communication development on schedule was observed in those aged 11-12 months and 15-16 months. High proportions of on-schedule development were also observed in children aged 3-4 months (87.5%), 17-18 months (85.7%), and 26-28 months (82.4%). Children aged 1-2 months showed a higher need for intervention.

Conclusions: The majority of children exhibited communication development on schedule according to ASQ communication scores. However, approximately one-fifth of children raised concerns that warranted further evaluation. A higher percentage of children in the youngest age groups (1-2 months and 29-34 months) required professional evaluation, monitoring, or intervention.

## Introduction

Language delay is among the most common developmental disorders in early childhood, marked by a delay in the acquisition of expressive or receptive language skills relative to age-matched peers [1, 2]. It affects a substantial proportion of children under the age of five and, if unaddressed, can lead to serious cognitive, social, and emotional consequences [3, 4]. Because communication is fundamental to a child's interaction with the environment, delays in this area may impede academic preparedness, social integration, and overall quality of life [5, 6]. Speech and language disorders may involve difficulties with articulation, verbal expression, or language comprehension. They are generally classified into receptive language disorders, which impair a child's ability to understand spoken language, and expressive language disorders, which affect the ability to convey thoughts and ideas [7, 8].

Speech and language delays are globally prevalent, affecting an estimated 5% to 10% of children under five who considerably lag in language development [9, 10]. This figure increases to approximately 8% to 12% of preschoolers in the United States [11], and may be higher in low- and middle-income countries due to limited access to early screening and intervention [12].

Language delays often accompany other developmental conditions, including autism spectrum disorder (ASD), hearing impairment, and intellectual disability, complicating diagnosis and management [13]. While a Baghdad study found a significant association between primary speech-language delay and family history, no such association was found with child age, postnatal complications, or television viewing [14]. Further research indicates that a family history of speech-language delay and male gender are significant risk factors, while birth order, family size, childhood illnesses, and parental education levels are less frequently reported as such [14, 15]. Research also suggests that parental reports are a reliable and effective method for assessing language abilities [16].

Parental communication considerably influences children's language development. Parent-child interactions affect not only language skills but also social and emotional development, although each child's unique genetic background also contributes to their overall development, personality, and social adaptability [17]. A study of 1,235 preschoolers in Saudi Arabia's Eastern Province found a 24.5% prevalence of language delay, associated with younger age (particularly in three-year-old boys) and a family history of language delay. Furthermore, limited parent-child interaction and excessive screen time were significantly associated with delayed language development [18]. These findings underscore the importance of early detection and intervention. The consequences of untreated language delay are far-reaching, affecting not only the child but also their families and society, potentially leading to academic difficulties, low self-esteem, and social isolation [19]. Parental stress and anxiety regarding their child's future are common, alongside the considerable healthcare and educational costs associated with delayed interventions [20, 21]. In Saudi Arabia, childhood language delay is increasingly recognized as a public health concern. Alzahrani et al. reported that 45.5% of parents perceived language delays in their children, with a higher prevalence (53.1%) among children aged three to five years [22].

The increasing global and local recognition of language delay as a developmental risk factor necessitates assessing its prevalence and associated risk factors. This study aims to determine the prevalence of language delay among children under five in Taif, Saudi Arabia, and to identify associated sociodemographic and developmental factors to inform effective early screening and intervention strategies.

## Materials and methods

Study design and population

This cross-sectional observational study, conducted in Taif, Saudi Arabia, from August 2024 to January 2025, assessed the prevalence of language delay in children under five years of age. Participants were children with a clinical diagnosis of language delay. Those who did not complete the survey were excluded.

Sample size and data collection tools

Sample size was determined using the Raosoft Sample Size Calculator (Raosoft Inc., Seattle, Washington), based on Taif's estimated under-five population of 97,262, an expected 15% prevalence of language delay, a 95% confidence interval, and a 5% margin of error. While the calculated minimum sample size was 196, the study included 400 participants to ensure robustness.

Data collection was performed randomly using a hospital-based questionnaire containing multiple-choice questions, distributed among participants in different centers across Taif. Data were collected via an electronic survey administered to parents of eligible children after obtaining informed consent. The survey used the Ages and Stages Questionnaire (ASQ), designed for specific age groups, to assess language delays. It comprised two sections: a demographic section collecting information about the child’s age, gender, residence, and the respondent’s relationship to the child; and a developmental assessment section evaluating milestones and factors related to language development.

Data analysis

Data analysis was performed using IBM SPSS Statistics for Windows, Version 21 (Released 2012; IBM Corp., Armonk, New York), using two-tailed tests with an alpha level of 0.05. Descriptive analysis involved presenting frequency distributions and percentages for the study variables, including participants' demographic data and the prevalence of language delay.

Ethical considerations

This study was approved by the Research Ethics Committee of Armed Forces Hospitals (Approval No. 2024-786). Participants were informed about the study objectives at the beginning of the survey, and informed consent was obtained thereafter.

## Results

Table 1 presents the sociodemographic characteristics of the under-five children assessed for speech delay in Taif, Saudi Arabia. Males comprised 176 (64.2%) of the sample, with females representing 98 (35.8%). The largest age group was 57-66 months, accounting for 50 children (18.2%), followed by 35-38 months with 21 children (7.7%), and 51-56 months with 20 children (7.3%).

**Table 1:** Sociodemographic characteristics of the children

Variable	Category	N	%
Gender	Female	98	35.8
	Male	176	64.2
Age of the child	1–2 months	8	2.9
	3–4 months	8	2.9
	5–6 months	14	5.1
	7–8 months	6	2.2
	9 months	7	2.6
	10 months	7	2.6
	11–12 months	7	2.6
	13–14 months	12	4.4
	15–16 months	3	1.0
	17–18 months	7	2.6
	19–20 months	7	2.6
	21–22 months	9	3.2
	23–25 months	16	5.8
	26–28 months	17	6.2
	29–31 months	18	6.6
	32–34 months	7	2.6
	35–38 months	21	7.7
	39–44 months	16	5.8
	45–50 months	14	5.1
	51–56 months	20	7.3
	57–66 months	50	18.2

The classification of communication stages among children assessed for speech delay was determined using ASQ scores based on age group. The analysis revealed that the majority of children, 169 (61.7%), demonstrated “communication development that appeared to be on schedule.” Meanwhile, 49 (17.9%) “required additional learning activities and ongoing monitoring,” and 56 (20.4%) were identified as “potentially needing further professional assessment” (Table 2).

**Table 2:** Communication stages classification

Stages	N	%
Child's development appears to be on schedule	169	61.7
Provide learning activities and monitor	49	17.9
Further assessment by a professional may be needed	56	20.4

Table 3 details the communication stage analysis by age group. Children aged 11-12 months and 15-16 months showed the highest proportion, 7 (100%) and 3 (100%), respectively, of age-appropriate communication development. High proportions were also observed in the 3-4 month group, 7 (87.5%); 17-18 month group, 6 (85.7%); and 26-28 month group, 14 (82.4%). Conversely, younger children, especially those aged 1-2 months, exhibited a greater need for intervention, with only 25% showing age-appropriate development, 50% requiring additional learning activities and monitoring, and 25% needing further professional assessment. Similarly, only 2 (28.6%) of 32-34-month-olds demonstrated age-appropriate development, with 3 (42.9%) requiring monitoring. Among 29-31-month-olds, 8 (44.4%) were on schedule, while 6 (33.3%) needed professional assessment. Older children (57-66 months) also showed a higher rate, 15 (30%), requiring further assessment. In contrast, children in the 39-44 month and 45-50 month age ranges displayed higher rates of age-appropriate development, 14 (87.5%) and 10 (71.4%), respectively, although some still required monitoring or further evaluation. Of note, the 39-44 month range was not explicitly detailed but inferred based on the provided data encompassing 39-50 months.

**Table 3:** Stages of communication based on the age of the child

Age of the child	Communication			P-value
	Child's development appears to be on schedule	Provide learning activities and monitor	Further assessment by a professional may be needed	
1–2 months	2 (25.0%)	4 (50.0%)	2 (25.0%)	0.045
3–4 months	7 (87.5%)	0 (0.0%)	1 (12.5%)	
5–6 months	11 (78.6%)	1 (7.1%)	2 (14.3%)	
7–8 months	2 (33.3%)	3 (50.0%)	1 (16.7%)	
9 months	5 (71.4%)	1 (14.3%)	1 (14.3%)	
10 months	3 (42.9%)	3 (42.9%)	1 (14.3%)	
11–12 months	7 (100.0%)	0 (0.0%)	0 (0.0%)	
13–14 months	7 (58.3%)	4 (33.3%)	1 (8.3%)	
15–16 months	3 (100.0%)	0 (0.0%)	0 (0.0%)	
17–18 months	6 (85.7%)	1 (14.3%)	0 (0.0%)	
19–20 months	3 (42.9%)	2 (28.6%)	2 (28.6%)	
21–22 months	7 (77.8%)	2 (22.2%)	0 (0.0%)	
23–25 months	12 (75.0%)	1 (6.3%)	3 (18.8%)	
26–28 months	14 (82.4%)	1 (5.9%)	2 (11.8%)	
29–31 months	8 (44.4%)	4 (22.2%)	6 (33.3%)	
32–34 months	2 (28.6%)	3 (42.9%)	2 (28.6%)	
35–38 months	10 (47.6%)	4 (19.0%)	7 (33.3%)	
39–44 months	14 (87.5%)	0 (0.0%)	2 (12.5%)	
45–50 months	10 (71.4%)	1 (7.1%)	3 (21.4%)	
51–56 months	11 (55.0%)	4 (20.0%)	5 (25.0%)	
57–66 months	25 (50.0%)	10 (20.0%)	15 (30.0%)	

Analysis of communication stages by gender showed that a greater percentage of girls, 67 (68.4%), demonstrated age-appropriate communication development compared to boys. Conversely, boys had slightly higher proportions requiring additional learning activities and monitoring, 32 (18.2%), and further professional assessment, 42 (23.9%), than girls, 17 (17.3%) and 14 (14.3%) respectively (Table 4).

**Table 4:** Stages of communication based on the gender of the child

Gender of child	Communication			P-value
	Child's development appears to be on schedule	Provide learning activities and monitor	Further assessment by a professional may be needed	
Female	67 (68.4%)	17 (17.3%)	14 (14.3%)	0.138
Male	102 (58.0%)	32 (18.2%)	42 (23.9%)	

## Discussion

Developmental delays occur when children fail to meet expected milestones. Speech delay, a common concern, involves language acquisition that is significantly slower than that of peers [1]. This can manifest as articulation difficulties, limited vocabulary, or trouble understanding and using language effectively [2, 3]. Although some children catch up naturally, persistent delays can negatively impact academic, social, and emotional development, underscoring the need for early identification and intervention [4, 5]. This study investigated communication development and the prevalence of speech delay among children under five in Taif. Although most children showed age-appropriate communication development based on ASQ scores, a substantial proportion (approximately 20%) required further assessment. The youngest age groups (1-2 months and 29-34 months) showed the highest need for intervention, monitoring, or professional assessment, highlighting the vulnerability of young children to communication delays and the need for proactive interventions at these critical developmental stages [6, 7]. The lower proportion of 29-34-month-olds with age-appropriate communication may reflect increased parental awareness or genuine developmental concerns, underscoring the need for targeted public health initiatives and educational support.

Previous research indicates a subtle but consistent gender difference in early speech and language development, with girls generally exhibiting a slight advantage over boys [8-10]. This advantage often manifests as larger vocabularies, more complex sentence structures, and superior grammatical skills in girls at younger ages [8]. Several factors may contribute, including biological influences such as variations in brain development and hormonal factors [11, 12], with some studies suggesting faster brain maturation in language processing areas in girls [13, 14]. Environmental factors, such as parental interaction styles, may also play a role. For instance, studies suggest parents often engage in more verbal interaction with infant daughters than sons, potentially providing girls with greater language exposure [15-17]. Although Alsaadi et al.'s study in Dubai and the Northern Emirates identified media exposure and social interaction as risk factors for language delay [18], it did not specify gender-based differences. However, these factors could possibly affect boys and girls differently. It is important to note that these are general trends, and individual variations are significant. While girls, on average, may acquire language skills slightly earlier, many boys develop language at the same pace or even earlier than their female peers [19]. The observed gender differences are subtle and should not be generalized to individual children. Wilson et al. highlighted the difficulty of predicting language delay based only on risk factors, including gender [20]. Moreover, these gender-based differences tend to lessen over time. The gap in language skills typically narrows as children age, with most achieving similar proficiency by school age, regardless of gender [21]. While understanding these trends is valuable for research and educational purposes, it is important to avoid perpetuating stereotypes and causing unnecessary parental anxiety. Each child develops at their own pace, and it is important to focus on individual needs rather than gender comparisons to promote healthy development.

Alzahrani et al. reported a 45.5% prevalence of speech delay among Saudi children under seven, significantly higher in 3-5-year-olds and males, with a family history of communication disorders, hearing or motor impairments, and autism spectrum disorder (ASD) identified as significant risk factors [22]. Choudhury and Benasich similarly found that a family history of speech and language disorders increased the risk of delayed speech development [23]. Specific genetic syndromes, such as Down syndrome and Fragile X syndrome, are also associated with speech delays [24, 25]. Auditory processing difficulties, including difficulty hearing speech sounds, significantly hinder language acquisition [26]. Even mild or intermittent hearing loss, such as that resulting from recurrent ear infections, can affect speech development. Conditions affecting brain development, such as cerebral palsy or ASD, can also impact speech and language development [27, 28]. Premature birth and birth complications, including subdural hematoma, may increase the risk [29]. Oral structural abnormalities of the mouth, tongue, or palate can affect speech sound production, leading to articulation difficulties [30, 31]. Furthermore, limited exposure to language during early childhood, whether due to limited social interaction or exposure to multiple languages, can hinder speech development [32]. Research indicates a correlation between socioeconomic status and speech delay, with access to quality childcare, healthcare, and educational resources influencing language development [33, 34]. Premature birth is associated with developmental delays across multiple domains, including speech and language [35]. Chronic illnesses or prolonged hospitalizations can also impact overall development, including speech. Speech delay may sometimes be part of a broader developmental delay affecting multiple areas of functioning [36]. Speech delay is often multifactorial, resulting from a complex combination of biological and environmental factors requiring comprehensive assessment to identify specific causes and guide intervention. Early intervention is crucial for maximizing a child's communication potential because early identification and support significantly improve long-term outcomes [37]. A multidisciplinary approach, involving speech-language pathologists, audiologists, and other healthcare professionals, is often necessary to provide effective, individualized support for children with speech delays and their families [38].

Based on the findings of this research, several recommendations can be proposed. First, implementing routine, age-specific developmental screening programs at primary healthcare facilities could facilitate early identification and timely intervention for speech delays. Second, increasing awareness and implementing educational campaigns targeted toward parents and caregivers about normal speech development milestones, signs of delay, and the importance of early assessment is important. Third, developing gender-sensitive interventions that account for the higher prevalence of speech delay observed among males may improve outcomes. Fourth, additional research, particularly longitudinal studies with clinical assessments, should be performed to clarify risk factors, the progression of speech delay, and the effectiveness of early intervention strategies. Finally, healthcare professionals and educators should collaborate to provide comprehensive support systems, including resources for families with a history of developmental disorders, ASD, or hearing and motor impairments, because these factors were significantly associated with speech delays in this study.

This study has several limitations. The cross-sectional design prevents establishing causality or tracking the progression of speech delays. Parental self-report introduces potential bias and subjectivity, potentially leading to inaccurate reporting of speech issues. The lack of independent clinical confirmation of reported delays further limits the reliability of the findings. The geographically restricted sample (Taif, Saudi Arabia) limits the generalizability of results to broader populations and diverse settings. Finally, the study lacks data on key variables such as socioeconomic status, parental education, and home environment, which could influence speech development. These factors were not explored, potentially affecting the comprehensiveness of the findings.

## Conclusions

This cross-sectional study underscores the importance of supporting language development, especially in younger children. Interestingly, children aged 11-12 months and 15-16 months demonstrated an elevated rate of age-appropriate communication skills. High percentages were also observed in the 3-4 month, 17-18 month, and 26-28 month age groups, indicating that early intervention and continuous monitoring may be advantageous.

To better support children at risk of language delays, it is important to raise public awareness and strengthen collaboration among educators. Establishing comprehensive support systems is especially important for families with a history of developmental disorders, ASD, or hearing and motor impairments, which were identified in our study as being associated with speech delays. Through collective efforts, we can create an environment that supports healthy language development for all children.
